# The opacity myth: A response to Swofford & Champod (2022)

**DOI:** 10.1016/j.fsisyn.2022.100275

**Published:** 2022-06-19

**Authors:** Geoffrey Stewart Morrison, Nabanita Basu, Ewald Enzinger, Philip Weber

**Affiliations:** Forensic Data Science Laboratory, Aston University, Birmingham, UK; Forensic Evaluation Ltd, Birmingham, UK; Forensic Data Science Laboratory, Aston University, Birmingham, UK; Eduworks Corporation, Corvallis, OR, USA; Forensic Data Science Laboratory, Aston University, Birmingham, UK; Forensic Data Science Laboratory, Aston University, Birmingham, UK

**Keywords:** Forensic inference, Statistical model, Machine learning, Artificial intelligence, Understanding

## Abstract

Swofford & Champod (2022) FSI Synergy article 100220 reports the results of semi-structured interviews that asked interviewees their views on probabilistic evaluation of forensic evidence in general, and probabilistic evaluation of forensic evidence performed using computational algorithms in particular. The interview protocol included a leading question based on the premise that machine-learning methods used in forensic inference are not understandable even to those who develop those methods. We contend that this is a false premise.

Letter to the Editor

Swofford & Champod [1] reports the results of semi-structured interviews conducted with three anonymous individuals from each of the following groups of stakeholders: laboratory managers, prosecutors, defence attorneys, judges, academic scholars. The interviewees were asked their views on probabilistic evaluation of forensic evidence in general, and probabilistic evaluation of forensic evidence performed using computational algorithms in particular.

The issue we wish to raise here is the use of a leading question in the interview protocol that may have led to bias in the interviewees’ stated opinions, and that promotes what we call *the opacity myth*. The questions was:

Many modern computational algorithms are based on artificial intelligence and machine learning (AI/ML) methods, which are often “black boxes” even to their developers irrespective of the availability of the source-code. What is your opinion about the use of these algorithms in forensic science for court purposes?

The question incorporates the premise that machine-learning methods used in forensic inference are not understandable even to the developers of machine-learning-based forensic-inference systems. This premise is repeated several times in the Swofford & Champod [1] article itself.

As developers of forensic-inference systems that make use of statistical-modelling and machine-learning methods, e.g., [2], [3], we contend that this is a false premise.^1^ We do understand how the methods we use work. They are technology. They are not magic.

One could pursue an *argumentum ad extremis* and reach a level at which there are things that developers do not understand, but this would become a philosophical debate about the meaning of “understand”, and would have no practical relevance.

Contrary to a claim of opacity made in §1 of Swofford & Champod [1], “although algorithmic tools generally possess remarkable potential to provide advanced scientific capabilities and promote more objective foundations to the evaluation of forensic evidence, they often do so at the cost of transparency and explainability”, forensic-evaluation systems that calculate likelihood ratios using relevant data, quantitative measurements, and statistical models/machine-learning algorithms are actually paragons of transparency [4], [5]. The data and software can be shared with other practitioners, and the algorithms implemented in the software can be exactly described. In addition, such systems can be (and should be) empirically calibrated and validated under conditions that reflect those of each case to which they are applied [6]. In contrast, what is opaque, and what is practically difficult to empirically calibrate and validate (and hence seldom is), is human perception and subjective judgement based on training and experience [7], [8].

We think that the opacity myth is related to a broader prejudice against the use of statistics and machine learning, and we would particularly commend the discussion of this topic in Swofford & Champod [9]. We think that the term *artificial intelligence* is an unfortunate buzzword whose connotations contribute to this prejudice. For many people, we think that *artificial intelligence* conjures up science-fiction stories involving Frankensteinian machines. We would recommend that the term not be used in the context of forensic inference.

It is of course true that those who have not studied and have not gained experience implementing and using statistical-modelling and machine-learning methods will be unlikely to understand them. Triers of fact are therefore unlikely to understand forensic-inference systems that make use of statistical models and machine-learning algorithms. That triers of fact are unlikely to understand evidence in a particular field unaided is the reason why expert witnesses with knowledge, training, and experience in that field are called to testify. But what is it that they must testify about? §5 of Swofford & Champod [1] claims that “algorithms need to be understandable and explainable to lay fact-finders”. We argue that this is not the case, and agree with Curran [10] that “As an expert presenting evidence to the court, I have an obligation to use the best scientific methods available to me, not the ones that are the easiest to explain.” US Federal Rule of Evidence 702 combined with the *Daubert* trilogy of Supreme Court rulings^2^ and England & Wales Criminal Practice Directions 19A^3^ both identify method validation under conditions relevant for the case as a consideration for admissibility, but neither identifies as considerations for admissibility either the explainability of an expert witness's methods or the understandability of those methods for the trier of fact. What provides the warrant for whether the trier of fact should or should not trust the output of a forensic-inference system is not understanding by the trier of fact of the methods that constitute that system, but validation of that system. What the trier of fact needs to understand are: first, whether the system has been validated under conditions sufficiently representative of those of the case under consideration; and, second, whether the results of that validation indicate that the system works sufficiently well under those conditions.

## Disclaimer

All opinions expressed in the present paper are those of the authors, and, unless explicitly stated otherwise, should not be construed as representing the policies or positions of any organizations with which the authors are associated.

## Declaration of competing interest

The authors declare that they have no known competing financial interests or personal relationships that could have appeared to influence the work reported in this paper.

## Author contributions

Morrison: Writing - Original Draft, Writing - Review & Editing. Other authors: Writing - Review & Editing.

## Footnotes

1 Contrary to the impression that one might get from reading Swofford & Champod [1], there is no sharp dividing line between what constitutes a statistical model and what constitutes a machine-learning algorithm.

2 William Daubert et al. v Merrell Dow Pharmaceuticals Inc., 509 US 579 (1993); General Electric Co. v. Joiner, 522 U.S. 136 (1997); Kumho Tire Co. v. Carmichael, 526 U.S. 137 (1999).

3 Criminal Practice Directions [2015] EWCA Crim 1569.
